# Allergic Rhinitis in Adults with Chronic Suppurative Otitis Media

**Published:** 2015-07

**Authors:** Shadman Nemati, Reza Jafari Shakib, Maryam Shakiba, Nematollah Araghi, Seyyede Zeinab Azimi

**Affiliations:** 1 *Sinonasal Diseases Research Centre, Amiralmomenin Hospital, Guilan University of Medical Sciences, Rasht, Iran.*; 2 *Department of Immunology, Faculty of Medicine, Guilan University of Medical Sciences, Rasht, Iran.*; 3 *Department of Epidemiology, School of Public Health, Shahid Beheshti University of medical Sciences, Tehran, Iran.*; 4 *Department of Otolaryngology, Amiralmomenin Hospital, Guilan University of Medical Sciences, Rasht, Iran.*

**Keywords:** Allergic, Hypersensitivity, Otorhinolaryngologic diseases, Otitis Media, Rhinitis, Suppurative, Skin test

## Abstract

**Introduction:**

Chronic suppurative otitis media (CSOM) is considered one of the most common causes of acquired hearing impairment in developing countries. CSOM is a multifactorial persistent inflammatory disease of the middle ear. A distinct pathophysiologic mechanism linking allergic rhinitis (AR) and CSOM remains to evolve. The purpose of this study was to investigate the association between AR and CSOM in adults.This was a case-control study.

**Materials and Methods:**

The subjects were 62 adults (23 male, 39 female) with established CSOM and 61 healthy controls. CSOM was diagnosed when there was a history of chronic (persisting for at least 3 months) otorrhea, accumulation of mucopurulent exudates in the external auditory canal or middle ear and/or perforated tympanic membrane on otoscopy. All participants were evaluated for the presence of AR by clinical evaluation of allergic symptoms, and underwent a skin-prick test for 23 common regional allergens. Statistical analysis was performed using SPSS version 16.

**Results:**

The prevalence of clinical rhinitis (allergic and non-allergic) was significantly higher among the cases compared with controls (62.5% vs. 37.5%, P=0.02). The prevalence of AR (proven by positive skin-prick test) was also significantly higher among affected adults than controls (24.6% and 13.8%, respectively). Adjusting for age, a logistic regression model showed that there was a significant difference between the two groups. Patients with AR and non-AR were at 3.27- (95% CI=1.15–9.29; P=0.036) and 2.57-(95% CI=1.01–6.57; P=0.048) fold increased risk of developing CSOM, respectively, compared with healthy individuals.

**Conclusion:**

The study showed a higher prevalence of AR in CSOM patients than in controls. It may be valuable to evaluate and control this factor in these patients.

## Introduction

Chronic supportive otitis media (CSOM) is a major problem facing health systems around the world. The condition is characterized by persistent inflammation of the middle ear and mastoid cavity associated with otorrhea through a perforated tympanic membrane, persisting for more than 6 weeks (1). The worldwide burden of CSOM is 65–330 million people, and approximately 60% suffer from clinically significant hearing impairment (2,3).

The pathogenesis of CSOM is considered multifactorial, and most patients with CSOM have a history of recent acute onset of otitis media, risk factors associated with acute otitis media, or otitis media with effusion. The pathogenesis is thought to include Eustachian tube (ET) dysfunction, immature or impaired immunologic status, upper respiratory allergy, familial predisposition, presence of other siblings, male sex, passive smoking and other factors (4,5). However, the risk factor for CSOM have not yet been fully defined (6).

With the prevalence of 10–30%, allergic rhinitis (AR) is the most common allergic disorder. AR occurs in association with a number of other disorders, principally sinusitis, asthma, allergic conjunctivitis , and atopic dermatitis (7-10). Studies show an increased prevalence of migraine headache in patients with AR (11,12). A relationship between AR and CSOM has been postulated for years. Evidence of a common pathophysiologic mechanism linking these two diseases continues to evolve (13). Because of the close anatomical relationship between ET and the nasopharynx, allergic disorders such as AR may cause ET dysfunction by inflammation and swelling in this region (14,15), and some studies have shown that an allergic challenge causes ET obstruction. Analysis of inflammatory mediators indicates that the mucosa of the middle ear can respond to antigens in the same way as does the mucosa of the lower respiratory tract (16). Although a definitive causal relationship between AR and CSOM remains to be demonstrated, a number of studies support the plausibility of this link (5,15,17,18).

Despite the existence of a few studies, there remains controversy about the relationship of AR and CSOM, and more studies are needed relating to the prevalence and role of allergy in the pathogenesis of CSOM (5,13,15,17,18). Therefore, we carried out the current study to investigate the association between allergic rhinitis and CSOM in a population of adult patients referring to the ENT-HNS University Hospital in Rasht, the most populated city in the north of Iran.

## Materials and Methods

In a case-control study, 62 patients who were candidates for tympanoplasty and mastoidectomy due to established CSOM were chosen, as well as 61 controls. The controls were selected from patients who were referred to the same hospital for minor head and neck trauma, with no history of CSOM or ear symptoms.

One of the cases and three of the controls were excluded from the study due to an inability to discontinue current medication or the presence of dermographism.

All subjects were examined by ear, nose and throat (ENT) specialists, and a thorough medical history and physical examination including anterior rhinoscopy and otoscopy, were performed.

The study was performed in Amiralmo- menin Educational Hospital and ENT Research Center of Guilan University of Medical Sciences (GUMS) in Rasht, Iran. Written informed consent was obtained from all the participants. The study protocol was approved by the ethics committee of GUMS.

CSOM was diagnosed when there was a history of chronic (persisting at least 3 months) otorrhea, accumulation of mucopurulent exudates in the external auditory canal or middle ear and/or perforated tympanic membrane on otoscopy.

AR was defined as the presence of signs and symptoms of clinical rhinitis, including watery anterior rhinorrhea, nasal obstruction or congestion, nasal pruritus, and sneezing, especially paroxysmal (according to standard questionnaire (19)). Post-nasal drip, pallor and swelling of the nasal and turbinate mucosa not due to a recent common cold could improve the clinical diagnosis. Patients with two or more of the mentioned suggestive symptoms for more than 1 hour on most days were clinically diagnosed as having AR (19). Clinical rhinitis was then confirmed by a positive skin-prick test (SPT). The diagnosis of AR was performed by a separate ENT specialist who was blind to the otologic situation of the patients.

All subjects underwent a SPT for 23 common allergens (AllergoPharma Products, Reinbeck, Germany) relevant in the north of Iran by a single immunologist, who was blind to both the otologic and rhinologic situation of participants. Allergens included six types of grass, four weeds, nine trees, two mites, cat allergen and Cladosporium.

The positive control was histamine hydrochloride (10 mg/mL) and the negative control was diluent (AllergoPharma). The mean wheal size was evaluated after 15 minutes, and SPT was determined as positive when observing a wheal with a mean diameter at least 3 mm larger than wheals at the site of the negative control. All subjects who were pregnant or had a history of recent consumption of antihistamines, immunotherapy with a specific allergen, or dermographism were excluded from the study. A positive SPT result could confirm the robust clinical diagnosis of AR, and negative results were considered as non-AR.

All data were analyzed using SPSS version 16. The χ2 and Fisher exact tests were used to test the significance of the differences between the two groups. A p-value less

than 0.05 was defined as significant. Odds ratio and 95% confidence intervals were also calculated.

## Results

A total of 61 cases (22 male and 39 female) with a mean age of 37.1±14.3 years (range 15–70 years) and 58 controls (27 male and 31 female) with mean age of 28.3±11.7 years (range 15–70 years) completed the study. There was a statistically significant difference between the groups in terms of age (P=0.047). Among the 61 cases with CSOM, 26 (42.6%) patients had right ear, 25 (41%) had left ear, and 10 (16.4%) had bilateral involvement. The female–to-male ratio was 1.7:1, but the difference was not statistically significant. Thirty-seven (60.7%) patients had a history of CSOM from childhood (<18 years); others developed the disease in adulthood. The exact time of presenting CSOM symptoms for those who developed CSOM from childhood was unavailable.

The proportion of patients with clinical rhinitis (allergic and non-allergic) was significantly higher in the cases compared with the control group (62.5% vs. 37.5%, P=0.02). The prevalence of AR (i.e. clinical rhinitis with positive SPT) was 24.6% (n=15) and 13.8% (n=8) among cases and controls, respectively. However AR was more prevalent among patients with CSOM compared with controls, although the difference was not statistically significant (P=0.065) (Table.1).

Using a logistic regression model, after correcting for the age factor, the difference between the two groups became significant. Patients with AR and non-AR had a 3.27- (95% CI=1.15-9.29; P=0.036) and 2.57- (95% CI=1.01-6.57; P=0.048) fold increased risk of developing CSOM, respectively, compared with healthy individuals.Patients with a history of childhood CSOM were more likely to have AR than the control group (29.7% vs.13.8%, P=0.038).

**Table 1:** Distribution of findings on clinical examination and SPT^1^ in cases and controls

		**CSOM** 2 **N=61**	**Control** **N=58**	**P-value**
Atopy	Atopy with Rhinitis			
		15 (24.6%)	8 (13.8%)	0.28
	Without Atopy	45 (73.8%)	48 (82.8%)	
	Atopy without Rhinitis	1 (1.6%)	2 (3.4%)	
				
Rhinitis	Allergic Rhinitis	15 (24.6%)	8 (13.8%)	0.065
	Non-Allergic Rhinitis	20 (32.8%)	13 (22.4%)	
	Without Rhinitis	26 (42.6%)	37(63.8%)	
				
SPT	Positive Negative	16(26.2) 45(73.8)	10(17.2) 48(82.8)	0.23

1 .Skin Prick Test

2 . Chronic Suppurative Otitis Media

Among all participants with AR, 52.2% (n=12) had a posterior nasal drip, 34.8% (n=8) had lower turbinate hypertrophy, and 60.7% (n=14) had pallor and swelling of the turbinate mucosa. Indoor allergens, especially mites (dermatophagoides farina and dermatophagoides pteronyssinus) were the most prevalent allergens in both groups, while outdoor allergens such as grass pollen and weeds were less prevalent (Table. 2).

**Table 2:** Comparison of the frequency of the sensitivity to common allergens among case and control group

Common Allergens	CSOM^1^	Control
**Grasses** 2	1(6.25%)	1(10%)
**Trees І** 3	1(6.25%)	0(0%)
**Trees ІІ** 4	2(12.5%)	3(30%)
**Weed** 5	1(6.25%)	1(10%)
**Dermatophagoides farina**	12(75%)	9(90%)
**Dermatophagoides pteronyssinus**	8(50%)	6(60%)
**Cladosporium**	1(6.25%)	0(0%)
**Cat & dog epithelia**	1(6.25%)	0(0%)

1 . Chronic Suppurative Otitis Media

2 . Grasses (Orchard grass, Velvet grass, Rye grass, Timothy grass, Kentucky grass, Meadow grass)

3 . Trees І (Alder, Hazel, Poplar, Elm, Willow tree)

4 . Trees ІІ (Birch, Beech, Oak, Plane tree)

5 . Weed (Mugwort, Nettle, Dandelion, Engl. Plantain)

## Discussion

As CSOM is associated with recurrent attack of otitis media and allergy and contributes to chronic otitis media with effusion, it is plausible that allergy also contributes to CSOM. Previous studies have reported wide prevalence of AR in otitis media with effusion, ranging from 24–89% (14,20,21). There are a number of studies investigating the association of CSOM and allergy, but they are controversial and there is no well-established link between AR and CSOM (5,18,22-24). The findings of this study revealed that there is a relationship between CSOM and AR. This finding is congruent with the results of some previous studies (5,18,22), but is in contrast with the studies of Fliss et al. and Bakhshaee et al. (23,24). A possible explanation is that these differences may be due to different methods of evaluating AR. In Lasisi’s study, serum total immunoglobulin E (IgE) concentrations were regarded as allergic assessment tests (18), while recent investigations show that because of low sensitivity and specificity, serum total IgE level is not a reliable parameter for screening atopic diseases (25). Bakhshaee et al. reported a 29.41% prevalence of AR among adults with CSOM, which was higher than the reported prevalence in our study; however they used serum total IgE level as an assessment tool for allergy diagnosis. In this study, the diagnostic criteria for AR consisted of a positive SPT to at least one allergen and/or a high level of serum total IgE, as well as a positive clinical examination and a history for rhinitis. Total IgE levels higher than 100 IU/ml were considered as a complementary test in cases with an established history ofAR (24). 

Fliss and colleagues collected data during the children’s visit to the clinic by means of a structured interview with the parents using an appropriate questionnaire and by extracting from the records if necessary (23). Gorgulu et al. measured total and allergen-specific IgE levels, as well as blood eosinophil counts. Endoscopic evaluations of the rhinopharynx were performed as well. In addition, a positive reaction to at least one of the 20 regional aero-allergens was accepted alongside allergen-specific IgE tests or total IgE level >300 IU/ml or a positive rate of blood eosinophil count (5). Hong and colleagues performed allergy tests which included total IgE and multiple radioallergosorbent chemiluminescence assays to check for the presence of IgE-mediated hypersensitivity (22).

CSOM could be a complication of acute otitis media or otitis media with effusion, both of which are more prevalent in the early childhood (4). Also, AR more commonly develops before the age 20 years (26). To the best of our knowledge this is the first study in this region that categorized patients with CSOM into two groups according to the time of onset of disease. In our study, AR was more common in those who developed CSOM from childhood. In addition, most of the previous studies evaluating the relationship of AR and chronic otitis media had studied children. In contrast, our study, like the one conducted by Mion (17), studied adults. We could find no association between positive SPT per se and CSOM (Table 1), although we reported an association between AR and CSOM in our study. These findings are consistent with the results of a study by Caffarelli and colleagues (27) who demonstrated that only the presence of AR and not a positive SPT demands evaluation for otitis media with effusion. In our study, the prevalence of positive SPT in CSOM was also similar to the mentioned study (26.2% and 26.74%, respectively). Furthermore, in the present study, indoor allergens were more prevalent in the CSOM cases. This high prevalence may be due to the humid climate in the north part of Iran, as shown previously (28).

## Conclusion

AR is more frequent in CSOM patients, and may be a risk factor for CSOM. Avoidance of recognized allergens may reduce this risk and improve the outcome of surgical therapy. Further studies in this regard are warranted.
